# Diseases of the Nervous System

**Published:** 1905-05-27

**Authors:** 


### Progress in Medicine and Surgery.

### DISEASES OF THE NERVOUS SYSTEM.

**Spinal Cord Degenerations in Anæmia.**—Michell Clarke[1] has recorded five cases of degeneration of the spinal cord occurring in anæmia. The degeneration begins in the posterior columns and first in the cervical and upper dorsal regions of the cord; in more advanced cases the posterior columns are involved almost throughout the cord, though the lesion is most extensive in the region in which it began. These changes do not exactly map out special tracts, but are somewhat patchy in distribution, often varying at different levels for no apparent reason. The appearances in some instances suggest some relation to the vessels. In the cases in which the posterior column changes are most marked, a patch of degeneration is also to be seen in the crossed pyramidal tracts. It is more patchy and less regular even than the degenerations in the posterior columns. In the degenerated areas there is an increase in the neuroglial framework and the vessels often show greatly thickened walls; these changes are probably secondary to the parenchymatous degeneration of the nerve fibres. The most prominent clinical symptoms are paræsthesias of various kinds, numbness, tingling, pricking, appearing first in the feet and legs. Later, the hands and forearms may be affected, and the numbness makes fine movements difficult. Objective disturbance of sensation is not marked, but in the late stages may consist of some deficient appreciation of all forms of sensation over the lower extremities. After these sensory symptoms weakness in the legs may follow. A frequent and often troublesome symptom is twitching of the muscles, especially in the legs and at night. The deep reflexes are present, ankle clonus is not obtained, and the plantar reflex, flexor in type, is sometimes difficult to get. There is no ataxy and no incontinence of urine. These cases form a fairly distinct group, in which the changes occur in the cord as a result of anæmia and from forms of anæmia due to blood destruction. They do not occur in cases of chlorosis of however long standing. Possibly the parenchymatous degeneration is due to some toxin set free in the course of a pathological blood destruction. In pernicious anæmia lesions in the spinal cord of quite another kind may occur—numerous small hæmorrhages, chiefly in the grey matter, but the changes above described do not start from any lesions of this kind. The cases described differ from those called subacute combined sclerosis or diffuse degeneration of the cord, for in the latter class the degenerations are more precisely limited to the neuronic systems of the cord, or, in the more severe cases, much more extensive than in the above cases. Further, the anæmia is the first sign of illness, preceding any evidence of spinal cord lesion, and the clinical signs are never very pronounced. On the other hand, the microscopical details of the degenerative process appear to be similar in the two groups. There thus seem to be two distinct groups of cases—those of distinct anæmia with slight clinical signs of cord disease, and those of cord disease in which there is no anæmia, or, if it is present, it is late and consecutive to the appearance of symptoms of cord disease. Those cases which seem to combine the features of both groups, in presenting both profound anæmia and widespread cord degeneration, must be left provisionally in a third class pending further investigation.

**Locomotor Ataxy.**—The pains of tabes are divided, by Gowers,[2] into the following kinds:—(a) Those which are brief, commonly momentary, but succeeding each other after a short interval in the same place, commonly for hours, sometimes for days. These "lightning" pains may be either superficial or deep. They may seem to be on or just below the surface of any limb, especially the legs, or of the head or face; they make the skin tender to touch, even where sensibility to pain, as from the prick of a pin, is lost. It is probable that these superficial pains are due to a morbid action in the tactile nerves. Lightning pains may also be deeper, sometimes about the joints; they may be described as precisely like the pain of cramp, though no muscular contraction is present. They may be followed by a persistent increased sensitiveness in certain muscles whereby pain is caused by their contraction. (b) The pains of tabes may be prolonged—lasting hours or days in the same place; these are commoner in the trunk than the limbs. One form is superficial—a girdle sensation. It may be unilateral and may give rise to difficulties in diagnosis, *e.g.* it may be mistaken for intercostal neuralgia, renal or hepatic colic, sciatica. In rare cases the pains dominate the aspect of the disease, occurring when the knee-jerks are not lost and there is no ataxy. The evidence in such cases of their tabetic nature is their resemblance to the pains found in undoubted cases of tabes, the presence of past syphilis, the existence of Argyll Robertson pupils, in one case optic atrophy, and, in another, gastric crises. The diagnosis is difficult, but it should be remembered that, though other causes may produce pains resembling the deep dull pains of tabes, no other cause produces real lightning, momentary stabbing or flashing pains. Such cases rarely develop ataxy, but the pains persist. The source of tabetic pains is probably the distal nerve endings. For, in the case of the superficial pains, they are followed by tenderness of the skin, and in exceptional cases by trophic changes, and they do not occur in regions where the sense of touch is lost. The pains that may be felt in the trunk of a nerve, *e.g.*, in the sciatic, may be the result of changes in the nerve-endings in its sheath. Tabetic pains can often be lessened but seldom arrested. Local measures may relieve the superficial pains. For instance, chloroform sprinkled on lint, with oiled silk over it, usually gives some temporary relief. More lasting is the effect of a hypodermic injection of cocaine, one-quarter to one-third grain, at the upper part of the area in which the pains are felt. Such injections may also be used when deep pain is complained of in the feet, but not when the pain is in the substance of a limb. Phenacetin, antipyrin, and antifebrin are very useful unless the pains are of great intensity. Morphia may be necessary when the suffering is intense. The tendency to the occurrence of pains may be lessened by chloride of aluminium, five to ten grains, three times a day, and in some cases by salicylates.

**Epilepsy.**—Turner[3] has based a paper on the prognosis of epilepsy on a large number of cases observed at the National Hospital for the Paralysed and Epileptic and in the Epileptic Colony at Chalfont St. Peter. He finds that sex plays but little part in the general prognosis. A family tendency to either epilepsy or insanity, though offering no obstacle to the arrest of the seizures in favourable cases, materially increases the likelihood of the disease becoming confirmed and the supervention of dementia. A commencement of the disease in childhood or infancy is the least favourable for arrest of the fits and the most favourable for the production of the confirmed disease. The common type of epilepsy—that commencing during puberty—is the most favourable form, both as regards the arrest of the seizures and the absence of mental infirmity. Adult epilepsy is unfavourable, but senile epilepsy is tractable. The earlier a case is brought under systematic treatment the more hopeful the prognosis, and, conversely, there is a progressive tendency for the disease to become confirmed the longer it has lasted without definite treatment. The longer the interval between the attacks the greater the prospect of improvement or arrest. The more frequent the attacks the more common and profound is the associated dementia. The greatest percentage of arrests are found in cases of grand mal, the least in cases of petit mal occurring alone, and an intermediate number where both kinds of attacks occur. Long remissions of the attacks are not synonymous with the cure of the disease, they may occur under bromide treatment and come to an end with its cessation, or they may be broken by an accidental circumstance—a blow on the head, fall, or acute inflammatory disorder. In general it may be laid down that a cure has been established after an arrest of nine years, though a small percentage of cases do relapse after even that period. If this period of nine years be taken as a basis it may be said that 10 per cent. of cases are cured. It is found that 50 per cent. of cases in which the disease is arrested show this within one year of treatment. The presence or absence of stigmata of degeneration[4] also has an influence on the prognosis—by these are understood structural deviations from the normal arising during the period of development and brain growth—such as deformities of the face, nose, external ears, and hard palate. It was found that those cases in which the disease commenced between birth and the age of five years showed a larger percentage of stigmata than at other ages. Direct parental heredity to epilepsy or insanity is usually associated with more pronounced stigmata than collateral heredity. The absence of stigmata does not necessarily imply an early or favourable termination of the disease. The relation of stigmata and the mental state is particularly obvious—those epileptics who showed the slighter degrees of mental impairment only presented a nearly equal proportion with (21 per cent.) and without (19 per cent.) stigmata. On the other hand, of those in whom there was marked mental enfeeblement 53 per cent. exhibited and only 7 per cent. were free from stigmata. These facts are in favour of the view that the interparoxysmal mental condition in epilepsy is an integral part of the disease.

[1] Brain, Winter Number, 1904. [2] Brit. Med. Jour., Jan. 7, 1905. [3] Edin. Med. Jour., Dec. 1904. [4] Lancet, Feb. 18, 1905.

(*To be continued.*)

